# Unusual Anomalous Hall Effect in Two-Dimensional Ferromagnetic Cr_7_Te_8_

**DOI:** 10.3390/molecules29215068

**Published:** 2024-10-26

**Authors:** Yifei Ma, Rui Yao, Jingrui Wu, Zhansheng Gao, Feng Luo

**Affiliations:** 1 Tianjin Key Lab for Rare Earth Materials and Applications, Center for Rare Earth and Inorganic Functional Materials, School of Materials Science and Engineering, Nankai University, Tianjin 300350, China; mayifei0708@163.com (Y.M.); 2120220347@mail.nankai.edu.cn (R.Y.); 13110555220@163.com (J.W.); 2 Center for the Physics of Low-Dimensional Materials, International Joint Research Laboratory of New Energy Materials and Devices of Henan Province, Key Laboratory for High Efficiency Energy Conversion Science and Technology of Henan Province, School of Physics and Electronics, Henan University, Kaifeng 475004, China

**Keywords:** chemical vapor deposition, ferromagnetism, Cr_7_Te_8_ crystal, anomalous Hall effect, topological Hall effect

## Abstract

Two-dimensional (2D) materials with inherent magnetism have attracted considerable attention in the fields of spintronics and condensed matter physics. The anomalous Hall effect (AHE) offers a theoretical foundation for understanding the origins of 2D ferromagnetism (2D-FM) and offers a valuable opportunity for applications in topological electronics. Here, we present uniform and large-size 2D Cr_7_Te_8_ nanosheets with varying thicknesses grown using the chemical vapor deposition (CVD) method. The 2D Cr_7_Te_8_ nanosheets with robust perpendicular magnetic anisotropy, even a few layers deep, exhibit a Curie temperature (*T*_C_) ranging from 180 to 270 K according to the varying thickness of Cr_7_Te_8_. Moreover, we observed a temperature-induced reversal in the sign of the anomalous Hall resistance, correlating with changes in the intrinsic Berry curvature. Additionally, the topological Hall effect (THE) observed at low temperatures suggests the presence of non-trivial spin chirality. Our findings about topologically non-trivial magnetic spin states in 2D ferromagnets provide a promising opportunity for new designs in magnetic memory spintronics.

## 1. Introduction

Recent advances in condensed matter physics have unveiled a range of novel phenomena that emerge from the complex interactions between electron spin degrees of freedom and various geometric and topological effects [1]. A key concept in this area is Berry curvature (Ω), arising from the geometric phase that electronic wave packets acquire as they traverse a closed loop in parameter space [2]. Berry curvature in solid-state crystals act as an extra magnetic field that affects electron motion, leading to significant observable effects in the transport experiments of the Hall device [3]. Moreover, magnetic materials display a variety of manifestations of Berry curvature in both real and momentum space [4,5]. Specifically, non-collinear spin configurations generate Berry curvature in real space, which subsequently induces the THE in conducting electrons [6,7]. In contrast, the Berry curvature in momentum space significantly influences the occurrence of the AHE [4,8,9,10,11,12], potentially causing quantization and displaying non-linear dependence on the external magnetic field. However, a full comprehension of the intricate physics governing chiral spin textures is still lacking. Specifically, these textures impart an additional phase factor to charge carriers due to the emergent field they produce. This often results in a THE, separate from the intrinsic AHE. Therefore, THE is recognized as a reliable approach for the electrical detection of chiral spin configurations [13].

In recent years, significant interest has emerged in the field of 2D FMs [14,15], such as those in the Cr*_x_*Te*_y_* family [16], Cr_2_Ge_2_Te_6_ [17], Fe*_x_*GeTe*_y_* [18], Fe_3_GaTe_2_ [19,20], and CuCr_2_Te_4_ [21], due to their inherent high-temperature ferromagnetism, which makes them suitable for a variety of applications similar to traditional film or metamaterials [22,23]. Notably, these low-dimensional ferromagnetic systems, such as Cr*_x_*Te*_y_* heterostructures [24,25], bulk, or nanosheets (e.g., Cr*_x_*Te*_y_* [26,27,28,29], Bi-Cr_2_Te_3_ [30], Cu-Cr_7_Te_8_ [31]), illustrate the promising applications of these 2D FMs in chiral spintronics. In the Cr*_x_*Te*_y_* system, 2D Cr_7_Te_8_ nanosheets have garnered considerable attention due to their temperature-dependent AHE and gate tunable properties related to Berry curvature [29]. However, research on the potential presence of the THE in Cr_7_Te_8_ nanosheets is lacking. Exploring the presence of THE in the 2D Cr_7_Te_8_ nanosheets is important to illustrate the promising applications of 2D FMs in chiral spintronics.

Here, we employed a CVD system to synthesize ultrathin Cr_7_Te_8_ crystals (~125 μm) on mica substrates. We then systematically investigated the electro-transport properties of 2D Cr_7_Te_8_ nanosheets with a thickness range of approximately 4~20 nm. The *T*_C_ of these nanosheets is found to be as high as 270 K. The measurement of the AHE reveals that 2D Cr_7_Te_8_ nanosheets with varying thicknesses exhibit robust perpendicular magnetic anisotropy (PMA). More importantly, we observed a temperature-induced sign reversal in the AHE, which is linked to Berry curvature effects in the Cr_7_Te_8_ crystals. Additionally, we observe an extremely ultrathin Cr_7_Te_8_ nanosheet (~4.3 nm) with THE resistivity up to 0.26 µΩ·cm at AHE transition temperature (*T*_S_), which is comparable to other Cr*_x_*Te*_y_* families [32,33]. The observed THE signals in the ultrathin Cr_7_Te_8_ nanosheet can persisted up 74 K, indicating the persistence of a robust chiral spin texture in our thin Cr_7_Te_8_ nanosheet.

## 2. Results and Discussion

### 2.1. Crystal Growth and Structural Characterization

**Figure 1:**
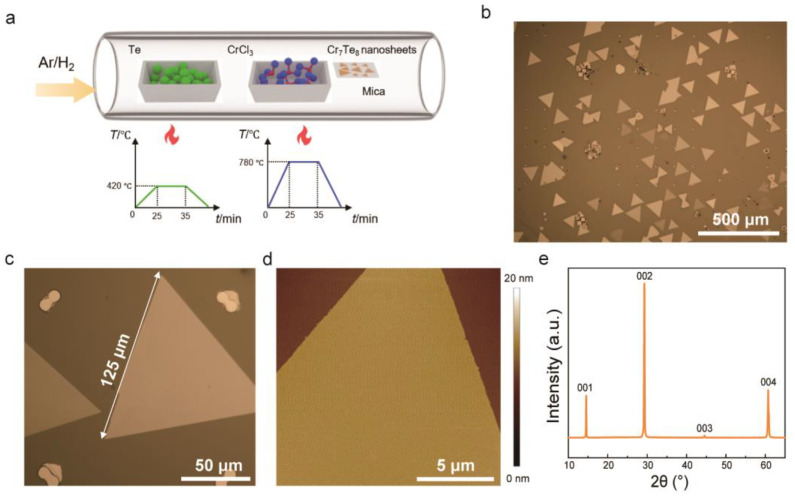
Synthesis and characterization of 2D Cr_7_Te_8_ nanosheets: (**a**) schematic diagram of the CVD system employed to grow Cr_7_Te_8_ nanosheets, where Te and CrCl_3_ sources were placed in separate zones with distinct heating processes; (**b**) typical optical microscopy (OM), illustrating the typical Cr_7_Te_8_ nanosheets; (**c**) OM image illustrating a Cr_7_Te_8_ nanosheet with an approximate domain size of 125 μm; (**d**) atomic force microscopy (AFM) image demonstrating the uniformity of the as-grown Cr_7_Te_8_ nanosheets with a thickness of ~3.65 nm (Figure S2); (**e**) XRD pattern of CVD-grown Cr_7_Te_8_ film.

**Figure 2:**
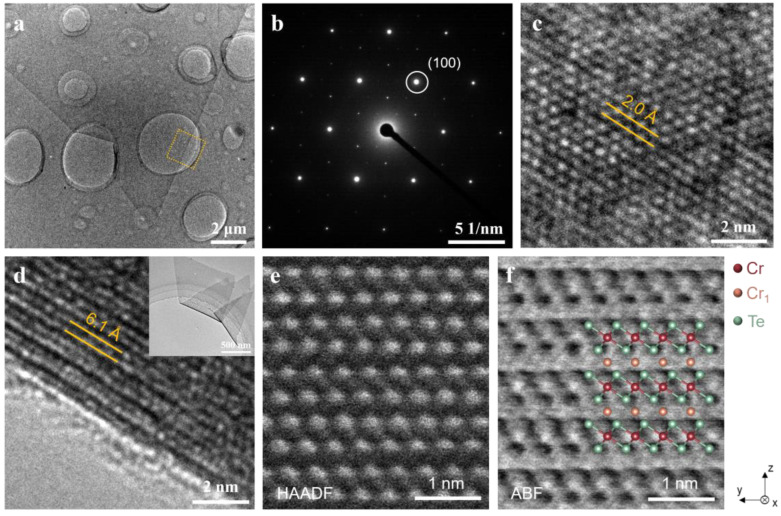
TEM characterization and atomic-scale structural analysis of the CVD-synthesized Cr_7_Te_8_ nanosheet: (**a**–**c**) TEM images of a typical Cr_7_Te_8_ nanosheet, oriented along the [001] zone axis and supported on a Cu grid, including the TEM image (**a**), SAED (**b**), and HRTEM (**c**), indicating that a hexagonal close-packed diffraction pattern was observed, and the lattice spacing of the (110) plane is about 2.0 Å. The yellow dashed rectangular box indicates the sampling location from which the measurements were obtained; (**d**) HRTEM image of the folded edge of a Cr_7_Te_8_ nanosheet (see inset), revealing a distinct layered spacing of about 6.1 Å; (**e**,**f**) cross-sectional TEM images of Cr_7_Te_8_ nanosheet on mica, aligned with [100] zone axis; (**e**) HAADF-STEM images display brighter contrast for Te (green ball) and darker contrast for Cr and Cr_1_ (red ball and orange ball), consistent with the NiAs-type Cr_7_Te_8_ crystal structure; (**f**) the corresponding ABF image.

Figure 1a depicts the growth of 2D Cr_7_Te_8_ nanosheets on mica substrates using a homemade CVD system. The mica substrate, with its atomically smooth surface and inherent layered structure, helps reduce the surface diffusion barrier energy of 2D Cr_7_Te_8_ nanosheets. In this setup, Te and CrCl_3_ powders were positioned in separate heating zones, i.e., upstream and downstream, each with distinct heating protocols. The reduction conditions were created by using Ar and H_2_ as the carrier gas (for more on the growth process, see Section 3). When the growth temperature varies between 680 °C and 780 °C, the morphology of the mica substrate deposition products transitions from hexagonal to triangular. Specifically, at lower temperatures, Cr_7_Te_8_ tends to exhibit a hexagonal appearance and forms thinner layers of crystals, while at higher temperatures, it is more likely to develop a triangular morphology, resulting in thicker samples (Figure S1). Typically, 2D Cr_7_Te_8_ nanosheets commonly display micrometer-scale triangular morphologies when the growth temperature 780 °C (Figure 1b). Through careful optimization of synthesis conditions, it is possible to achieve large (~125 μm) Cr_7_Te_8_ nanosheets with good homogeneity (*R*_q_~0.12 nm), as shown in Figure 1c,d. Improvements in surface roughness and crystal size are essential to exploring better performance and greater potential in various applications that demand high-quality nanoscale materials. Figure 1e presents the X-ray diffraction (XRD) pattern of Cr_7_Te_8_ films transferred to a glass substrate, which exclusively exhibits (00l) diffraction peaks. This observation indicates that the films grow primarily in the in-plane direction. Based on the Bragg equation, the interlayer spacing is calculated to be 6.08 Å (see more details in Table S1), which aligns with the folded edge of the Cr_7_Te_8_ nanosheet measured via high-resolution transmission electron microscopy (HRTEM), as shown in Figure 2d.

To achieve a thorough structural understanding of CVD-grown Cr_7_Te_8_ crystals, we employed TEM analyses. As illustrated in Figure 2a–c, the Cr_7_Te_8_ nanosheets were transferred onto Cu grids for the ab plane TEM, maintaining their characteristic triangular morphology (Figure 2a). Furthermore, the Cr_7_Te_8_ crystal exhibits a distinct hexagonal diffraction pattern, with the most prominent spots corresponding to the first-order diffraction in the (110) plane. This pattern effectively differentiates our Cr_7_Te_8_ sample from those that are sequentially embedded in the Cr layer, i.e., Cr_2_Te_3_, which would produce additional diffraction spots. Both HRTEM (Figure 2c) and selected area electron diffraction (SAED) reveal that the lattice spacing of the (110) plane is approximately 2.0 Å. As we know, HRTEM image of folded edges can provide an effective means of determining layer spacing for layered materials. As illustrated in Figure 2d, the Cr_7_Te_8_ nanosheet displays distinct layered lattice fringes with a spacing of approximately 6.1 Å, which closely matches the value of 6.08 Å obtained from XRD, as depicted in Figure 1e. Moreover, the crystal lattice of Cr_7_Te_8_ nanosheet shows significant differences compared to the conventional ratios of 1T-CrTe_2_ and CrTe [34,35] (see more details in Table S2). Hexagonal Cr_7_Te_8_ nanosheets, featuring a NiAs-type structure, can be distinguished by their alternating layers of Cr-poor and Cr-saturated regions. To examine the cross-sectional atomic arrangements along the [100] and zone axes, we utilized aberration-corrected scanning transmission electron microscopy (STEM) with high-angle annular dark-field (HAADF) and annular bright-field (ABF) imaging. Both imaging techniques utilize Z-contrast to differentiate between Cr and Te elements. From the HAADF image shown in Figure 2e, the Te columns are markedly brighter than the Cr columns, making it straightforward to distinguish between them. Nonetheless, the excessive brightness of the Te columns can make it challenging to identify layers with varying Cr content in an HAADF image. Due to its lower sensitivity to Z differences, ABF-imaging is often employed to identify vacancies or interfaces [36]. As illustrated in Figure 2f, the ABF images effectively differentiate between fully occupied Cr and intercalated Cr (Cr_1_).

### 2.2. Electromagnetic Transport Measurements

**Figure 3:**
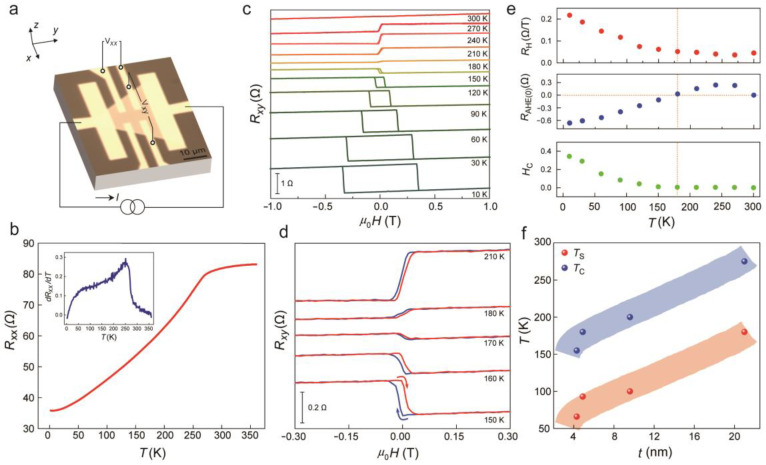
Electrical transport measurement of the CVD-grown Cr_7_Te_8_ nanosheets: (**a**) Colored OM image of a three-dimensional Hall device. (**b**) Longitudinal resistance *R_xx_*–*T* of a Cr_7_Te_8_ (~20 nm) hall device. The upper-left inset depicts the temperature dependence of the first derivative of *R_xx_*. (**c**) Temperature-dependent of Hall resistance (*R_xy_*) loops. The presence of ferromagnetism is indicated by the rectangular hysteresis observed in *R_xy_*. (**d**) The extracted *R_xy_*–*μ*_0_*H* curve from 150 to 210 K, highlighting the Hall resistance reversal occurring between 170 K and 180 K. (**e**) The temperature dependence of *R*_H_, *H*_C_, and *R*_AHE(0)_ for the Cr_7_Te_8_ hall device. (**f**) The Curie temperature *T*_C_ and transition temperature *T*_S_ of 2D Cr_7_Te_8_ nanosheets devices with varying thicknesses were determined by analyzing the first derivative of the *R_xx_*–*T* curve for *T*_C_ and examining the *R_xy_*–*T* loops for *T*_S_, identifying sharp slope changes and transition points, respectively. The blue and orange areas show the trend of *T*_C_ and *T*_S_ gradually increasing with the increase of thickness.

To confirm the ferromagnetism of 2D Cr_7_Te_8_ crystals, we conducted the AHE measurements using a Hall bar device (Figure 3a) via standard electron-beam lithography (EBL) process. Longitudinal resistance and transverse resistance signals were measured, underlying an *H_z_*. Figure 3b presents the *R_xx_*–*T* curve for a nanosheet approximately 20 nm thick, demonstrating a typical metallic resistance as a function of temperature. This indicates that the Pd/Au electrodes prepared via thermal evaporation provide good Ohmic contact at different temperatures. As illustrated in Figure 3c, we measured the anomalous Hall resistances for Cr_7_Te_8_ Hall devices in an external magnetic field. The measurements revealed that square hysteresis loops indicate that 2D Cr_7_Te_8_ crystals exhibit strong PMA. Additionally, the hysteresis loops persisted up to 270 K and vanished by 300 K, suggesting a relatively high *T*_C_ (>270 K, near room temperature). The *T*_C_ range of our 2D Cr_7_Te_8_ nanosheets at various thicknesses, compared to those reported for other 2D ferromagnet materials [17,19,21,31,34,35,37,38,39,40,41,42,43,44,45,46] (Figure S3), indicate a near room temperature *T*_C_. Based on Figure 3c, we extracted the Hall coefficient (*R*_H_), coercive field (*H*_C_), and Hall resistance at zero external magnetic field (*R*_AHE(0)_) as functions of temperature, respectively. Figure 3e illustrates that by performing a linear fit on the *R_xy_*–*μ*_0_*H* data within the high field region, we obtained a positive *R*_H_ at different temperatures. These results suggest that the Cr_7_Te_8_ nanosheets consistently exhibit hole-dominant electrical characteristics, a feature commonly observed in the Cr*_x_*Te*_y_* family. Additionally, the value of *H*_C_ gradually decreases and approaches zero at the *T*_S_, where the hysteresis loop almost vanishes. Beyond this point, *H*_C_ remains relatively small until it approaches zero again around 300 K. Note that the hysteresis loop becomes progressively narrower and eventually disappears near 180 K (Figure 3d). As the temperature rises, the sign of *R_xy_* reverses, indicating a transition temperature *T*_S_ near 180 K. Meanwhile, as the sample thickness decreases, the *T*_S_ shifts from approximately 170 to 40 K (Figure 3f). Similar reversal behavior in the AHE has been found in different Cr*_x_*Te*_y_* systems and is attributed to variations in Berry curvature within momentum space [47]. As an intrinsic mechanism, Berry curvature is crucial for understanding the origin of this reversal in AHE, particularly within metal-based ferromagnets with intermediate conductivity [1,3,4]. Typically, spin-degenerate bands in magnetic material will be divided into majority and minority bands at the Fermi level. The presence of SOC causes hybridization of these bands where they intersect. This band crossing, influenced by SOC, can result in a significant Berry curvature in the band structure, which, in turn, drives different physical transport phenomena, including the AHE. The sign of the AHE is thus directly associated with the distribution of the Berry curvature, which is quantified via integration across the electronic states occupied in momentum space [48]. A negative Berry curvature Ω at the Fermi level causes electrons to migrate towards the higher potential region, which yields a positive AHE (*R*_AHE_ > 0); conversely, a positive Ω results in electrons traveling to the lower potential side, leading to a negative AHE (*R*_AHE_ < 0). We suggest that the reduction in the thickness of the Cr_7_Te_8_ sample, along with a decrease in magnetization, modifies the Berry curvature distribution close to the Fermi level, contributing to a notable decrease in transition temperature *T*_S_ [29].

**Figure 4:**
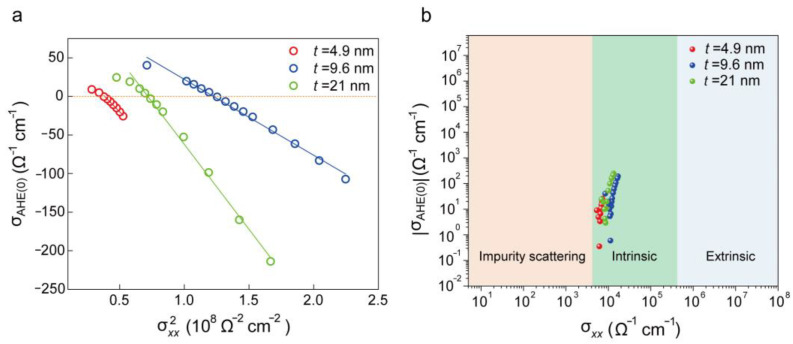
Analysis of the temperature-dependent sign reversal of the *R_xy_*. (**a**) Plot of $\sigma_{AHE(0)}$ versus the $\sigma_{xx}^{2}$ values at zero magnetic field for Cr_7_Te_8_ hall devices of varying thicknesses and temperatures. The lines represent linear fits demonstrating the proportional relationship $\sigma_{AHE(0)}\propto \;\sigma_{xx}^{2}$. (**b**) Plot of $\sigma_{xy}$ versus $\sigma_{xx}$ for Cr_7_Te_8_ nanosheets spanning the various AHE regimes from the impurity scattering mechanism (orange area) through the intrinsic (green area) and extrinsic regimes (blue area). The data points for all our Cr_7_Te_8_ samples, regardless of thickness, fall within the intrinsic region.

The AHE can generally be attributed to three independent mechanisms: skew scattering, side jump, and Berry curvature in momentum space [4]. To calculate the longitudinal resistivity $\rho_{xx}$ and the anomalous Hall resistivity $\rho_{AHE}$, one can use the anomalous Hall resistance $R_{AHE}$ and the longitudinal resistance $R_{xx}$. These resistivities are calculated using the following formulae: $\rho_{xx}=R_{xx}\cdot S/l$, $\rho_{AHE}=R_{AHE}\cdot S/l$, where S and l denote cross-sectional area and length of the Cr_7_Te_8_ conductive channel, respectively. Using the obtained $\rho_{xx}$ and $\rho_{AHE}$ values, the longitudinal conductivity $\sigma_{xx}$ and anomalous Hall conductivity $\sigma_{xy}$ were calculated using the following formulae: $\sigma_{xx}=\rho_{xx}/\left(\rho_{AHE}^{2}+\rho_{xx}^{2}\right)$, $\sigma_{xy}=\rho_{xy}/\left(\rho_{AHE}^{2}+\rho_{xx}^{2}\right)$. To reveal the physical origin of the AHE in Cr_7_Te_8_, whether intrinsic or extrinsic, we analyzed the dependence between the σ_AHE(0)_ and the corresponding $\sigma_{xx}$. Figure 4a illustrates the relationship between σ_AHE(0)_ and $\sigma_{xx}^{2}$ for Cr_7_Te_8_, showing a clear linear trend. This linear trend suggests that the AHE in Cr_7_Te_8_ is predominantly governed by the intrinsic contribution. Given the substantial SOC in Cr_7_Te_8_ and the intrinsic regime of the longitudinal conductivity (see Figure 4b), this strongly suggests that the intrinsic Berry curvature contribution dominates the AHE. This finding aligns with similar observations reported for Cr_1+δ_Te_2_ [47]. The temperature dependence of *R*_AHE(0)_ in Cr_7_Te_8_ nanosheet reveals two notable characteristics. First, when *T* < 170 K, *R*_AHE(0)_ is negative, indicating a Berry curvature Ω > 0. As the temperature rises, the sign of *R*_AHE(0)_ transitions from negative to positive around *T*_S_~170 K, reflecting a transition in Berry curvature from Ω > 0 to Ω < 0. Second, for temperatures above 300 K, *R*_AHE(0)_ approaches zero, reflecting a typical ferromagnetic to paramagnetic (FM-PM) transition. The observed sign reversal of *R*_AHE(0)_ across the *T*_S_ is likely due to a decrease in magnetization, which affects spin splitting and consequently modifies the Berry curvature distribution, leading to the sign change in *R*_AHE(0)_.

**Figure 5:**
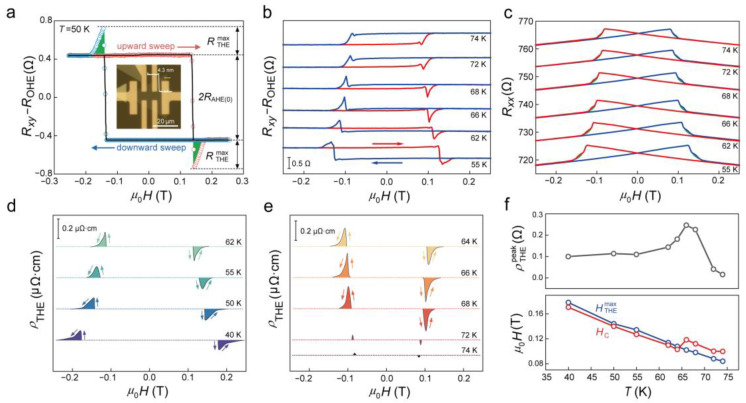
Investigation of potential topological hall effect in ultrathin Cr_7_Te_8_ nanosheet: (**a**) A detailed analysis of the Hall resistivity for the Cr_7_Te_8_ device (~4.3 nm) at *T* = 50 K with the subtraction of the OHE term. AHE term (indicated by the black lines) and THE term (highlighted by the green area) were shown. The AHE component is fitted by a $M_{0}tanh(\frac{H}{a_{0}}-H_{0})$ function, where *M*_0_, *a*_0_, and *H*_0_ are the fitting parameters. The inset is the OM image of the Cr_7_Te_8_ (~4.3 nm) hall device. (**b**) Magnetic field dependence of the subtracted transverse resistance from 55 to 74 K, clearly showing the *T*_S_ between 62 and 66 K. (**c**) Temperature dependence of *R_xx_* for the Cr_7_Te_8_ hall device, showing a pronounced hump feature up to 74 K (highlighted by olive filled areas), which correlates closely with the presence of THE. (**d**,**e**) Typical field dependence curves of the $\rho_{THE}$ at various temperatures (40~74 K). (**f**) Temperature dependence of the $\rho_{THE}^{max}$ (black line), along with the corresponding $H_{THE}^{max}$ (blue line) and $H_{C}$ (red line). All the different colored arrows indicate the magnetic field sweep directions.

In addition to temperature-dependent change in *R*_AHE_, another unusual phenomenon was observed in ultrathin Cr_7_Te_8_ nanosheets (~4.3 nm, shown in the inset of Figure 5a). Figure 5a presents the anomalous Hall resistances of Cr_7_Te_8_ at various temperatures, demonstrating ferromagnetic behavior. For clarity, linear OHE contributions have been subtracted. It is noted that an anomalous peak appears in the *R_xy_*–*μ*_0_*H* curves near the *H*_C_. These peaks appear irrespective of the sign of *R*_AHE_, even at *T*_S_~66 K. Two mechanisms are currently proposed to account for these abnormal Hall peaks: one is the bipolar two-component AHE model, which recent studies have shown to be ineffective for the Cr*_x_*Te*_y_* system [30,49]; the other is THE induced by non-coplanar spin textures [5]. Specifically, when conducting electrons travel between magnetic atoms in a non-coplanar spin configuration, the Berry curvature of the momentum space will generate an additional Hall signal as a virtual magnetic field, which is thought to be THE [50,51]. Furthermore, we have measured the longitudinal resistance *R_xx_* of the thin Cr_7_Te_8_ sample to further investigate this phenomenon. Figure 5b displays the field-dependent *R_xx_* between 55 and 74 K. All the *R_xx_*–*μ*_0_*H* curves exhibit butterfly-shaped loops, characteristic of ferromagnets exhibiting PMA. The maximum value of *R_xx_* occurs at *H*_C_, which is associated with the creation of the magnetic domain during magnetization reversal. In addition to this behavior, unusual magnetoresistivity humps appear close to *H*_C_ in the *R_xx_*–*μ*_0_*H* curves, highlighted by the green-filled areas in Figure 5c. The presence of these humps indicates a new scattering source affecting electromagnetic transport around *H*_C_ in our ultrathin Cr_7_Te_8_ sample. Notably, the region where these humps occur aligns precisely with the region where *R*_AHE_ exhibits changes, suggesting a shared origin for both phenomena. Two approaches are employed to extract the THE component: (1) utilizing a combination of linear fitting with a step function, $M_{0}tanh(\frac{H}{a_{0}}-H_{0})$, to subtract the AHE and OHE components [52]; and (2) extracting the difference in *R_xy_*–*μ*_0_*H* loops between sweeps in the upward and downward directions [53]. For this study, the first method was adopted, and the corresponding results can be seen in Figure 5d,e. These figures illustrate the THE component at different temperatures. At 66 K, THE achieves its peak at 0.1 T and then gradually diminishes to 0 in higher fields. The THE component remains detectable up to 74 K, though its intensity decreases with temperature. Beyond this temperature, only AHE is observed. It is noteworthy that similar THE characteristics with comparable critical temperatures can be replicated in another Cr*_x_*Te*_y_* system [30,32,49,54]. The maximal resistivity amplitude of the THE signal, $\rho_{THE}^{max}$, exhibits a notable peak of 0.26 μΩ·cm at 66 K and decreases continuously with both rising and falling temperatures (see Figure 5f). Notably, the observed $\rho_{THE}^{max}$ in Cr_7_Te_8_ is comparable to that reported in other systems [32,33]. The amplitude of $\rho_{THE}$ reflects the intensity of the interaction between the spin structure and electric current, indicating the effective magnetic field induced by the Berry phase in real space [48]. The significant reduction of the THE contribution to Hall resistivity, resulting from the high charge carrier density and decreased mean free path of electrons in metallic systems, is primarily due to the pronounced sensitivity of conductivity to disorder [55]. The temperature dependence of $H_{THE}^{max}$ is summarized in Figure 5f. The trend of $H_{THE}^{max}$, where $\rho_{THE}$ attains its maximum, aligns with *H*_C_, indicating that the spin chirality develops as the Cr atomic magnetic moment begins to reverse. Moreover, throughout the entire temperature range, while the reverse of the AHE, the sign of THE remains consistent, suggesting that the underlying mechanisms of THE and AHE are fundamentally different.

## 3. Methods

### 3.1. Growth and Characterization of Cr_7_Te_8_ Nanosheets

Two-dimensional Cr_7_Te_8_ nanosheets were synthesized on mica substrates in a homemade CVD system (JYG-1200-40-2-std, Shanghai Huiyi Technology Co., Ltd., Shanghai, China). Te (Alfa Aesar, 99.999%, 0.4 g) and CrCl_3_ powders (Alfa Aesar, 99.99%, 13 mg) were utilized as grown sources of chromium and tellurium, respectively. These powders were placed in separate heating zones, one upstream and one downstream. The mica substrates were positioned about 1 cm downstream from the CrCl_3_ heating zone. It is important to try to avoid Cr sources hydrolyzation before they are loaded into the furnace. After cleaning the furnace at least three times with high-purity argon (Ar, 99.99%), the Te and CrCl_3_ powders were heated at 420 °C and 680–780 °C for 15 min and held for 5 min for sample growth. The system pressure was maintained at 600 Torr during the growth process. Mixed gases of 5 s.c.c.m H_2_ and 45 s.c.c.m Ar were introduced to establish the appropriate atmosphere for the growth process. After the reaction, the system was cooled naturally to approximately room temperature.

The as-grown Cr_7_Te_8_ nanosheets were transferred onto copper girds for the TEM and the glass substrates for XRD characterization, respectively. The process is as follows: (1) the polystyrene (PS) solution was spin-coated at 3000 rpm for 60 s and then cured by baking at 90 °C for 15 min; (2) the edge of mica substrate was scratched and submerged in water for about 1 min (the PS film with as-grown Cr_7_Te_8_ nanosheets was easy to lift off the mica; (3) the PS film was transferred onto the desired substrate using PDMS, and the PS was removed using toluene.

The morphology and crystal structure of as-grown Cr_7_Te_8_ nanosheets were analyzed using OM (WY-910, VIYEE Photoelectric Co., LTD., Tianjin, China), AFM (Dimension Icon, Bruker corporation, Billerica, MA, USA), XRD (MiniFlex 600, Rigaku Corporation, Tokyo, Japan) and TEM (JEM-2800, JEOL LTD., Tokyo, Japan). Cross-sectional specimens were prepared via a focused ion beam (Helios 5 CX, FEI, Waltham, MA, USA) using a standard lift-out procedure. The conductive polymer (2000 rmp for 60 s) and Pt (200 nm) were deposited via e-beam for protecting the surface of the nanosheet from Ga ion damage. Atomic resolved cross-sectional specimens were performed on an aberration-corrected STEM (Titan cubed Themis G2 300, FEI, Waltham, MA, USA), operating at 200 kV.

### 3.2. Device Fabrication and Electromagnetic Transport Measurements

The as-grown Cr_7_Te_8_ nanosheets were fabricated into six-terminal Hall bar devices. The process is as follows: (1) location markers were defined via a standard UV photolithography process; (2) a standard EBL, deposition (thermal evaporation), and lift-off technique is employed to create the metal electrodes (Pd/Au, 5/30 nm) for the Hall bar structure; (3) silver and gold wires are used to connect the hall device to the sample holder. Electromagnetic transport measurements were performed in a PPMS system (Quantum Design Inc., San Diego, CA, USA). A perpendicular external magnetic field *H* was applied to the hall device plane. The Hall and magnetoresistance data were analyzed using symmetrization and antisymmetrization, respectively. Additionally, the *T*_C_ of the sample is determined via the temperature-dependent *R*_AHE(0)_–*T* curve extracted by *R_xy_*–*μ*_0_*H* at different temperatures, with its specific value located within the temperature range where *R*_AHE(0)_ begins to decrease to zero. The *T*_S_ of the sample is determined from the *R*_AHE(0)_–*T* curve, corresponding to the temperature at which the *R*_AHE(0)_ changes sign from negative to positive.

## 4. Conclusions

In summary, we successfully grew 2D Cr_7_Te_8_ ferromagnets with a Curie temperature close to room temperature using CVD technology. The Hall measurements clearly demonstrate that the perpendicular ferromagnetic order is maintained even in atomically ultrathin Cr_7_Te_8_ nanosheets (~4.3 nm). Moreover, an unusual anomalous Hall effect was observed at different Cr_7_Te_8_ nanosheets thicknesses. On one hand, the AHE exhibits a temperature-dependent sign-reversal as the Cr_7_Te_8_ nanosheet thickness changes, which is likely attributed to variations in the intrinsic Berry curvature. On the other hand, a significant THE, characterized by an anomalous Hall resistivity of 0.26 μΩ·cm, is observed at low temperatures in thin Cr_7_Te_8_ nanosheets, suggesting the presence of non-trivial spin chirality. Our study offers a valuable framework for investigating the connections between the magnetism, spin texture, and intrinsic Berry curvature in 2D FMs, and contributes to both fundamental research and the advancement of magnetoelectronic and spintronic technologies.

## Data Availability

Data presented in this study are available on request from the corresponding author.

## Supplementary Materials

The following supporting information can be downloaded at https://www.mdpi.com/article/10.3390/molecules29215068/s1, Figure S1: Morphology and thickness regulation of 2D Cr_7_Te_8_, Figure S2: Thickness testing and roughness analysis of Cr_7_Te_8_ nanosheets, Figure S3: *T*_C_ and thickness comparison for various 2D ferromagnet materials; Table S1: Layer spacing of Cr_7_Te_8_ calculated via the Bragg equation, Table S2: Comparison of crystal lattice constants of Cr_7_Te_8_ nanosheet with 1T-CrTe_2_ and CrTe.
