# Supplementary material for: Unusual Anomalous Hall Effect in Two-Dimensional Ferromagnetic Cr7Te8
Source: Molecules. 2024 Oct 26;29(21):5068. doi: 10.3390/molecules29215068 (PMC11547251; doi:10.3390/molecules29215068)
Supplement: Supplementary file 1 [file molecules-29-05068-s001.zip › molecules-3222509-supplementary.pdf]

Supplementary Information

Unusual anomalous Hall effect in two-dimensional ferromagnetic Cr_7Te_8

Yifei Ma[1], Rui Yao[1], Jingrui Wu[1], Zhansheng Gao[2*], Feng Luo[1*]

[1]Tianjin key lab for rare earth materials and applications, Center for rare earth and inorganic functional materials, School of materials science and engineering, Nankai University, 300350 Tianjin, China.

[2]Center for the Physics of Low-Dimensional Materials, International Joint Research Laboratory of New Energy Materials and Devices of Henan Province, Key Laboratory for High Efficiency Energy Conversion Science and Technology of Henan Province, School of Physics and Electronics, Henan University, Kaifeng 475004, China

*Correspondence to: zsgao@henu.edu.cn; feng.luo@nankai.edu.cn

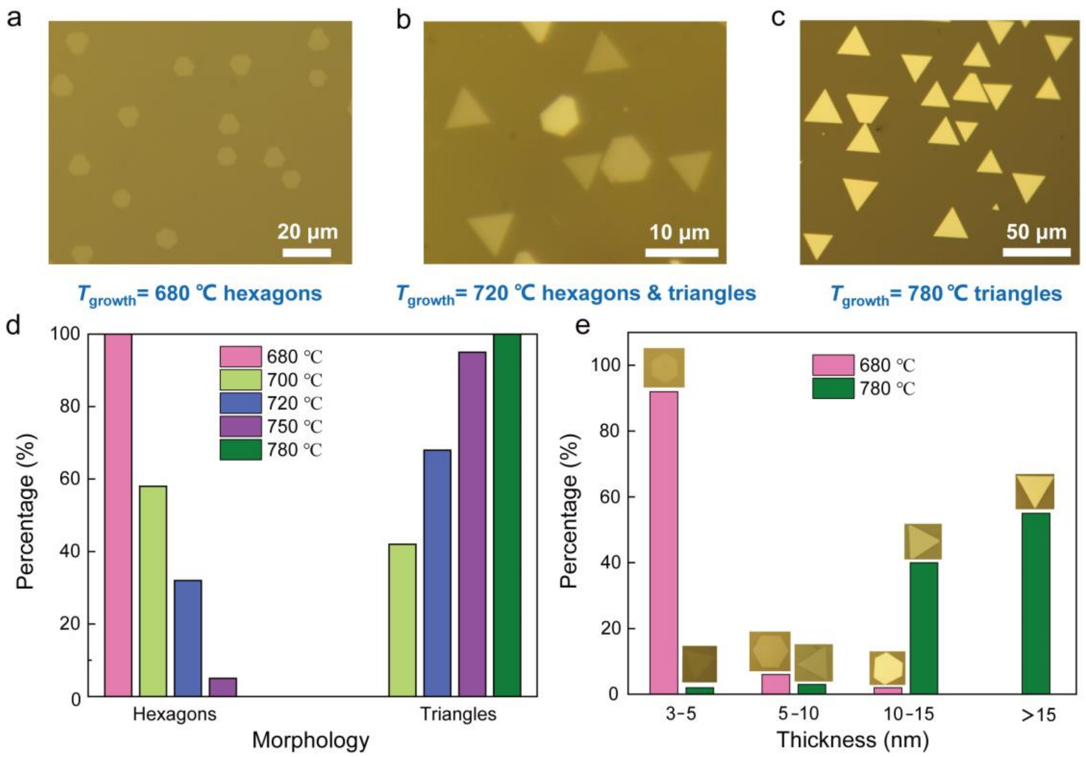

Figure S1. Morphology and thickness regulation of 2D Cr_7Te_8: (a–c) typical 2D Cr_7Te_8 optical images at different growth temperatures; (d) the proportion distribution of triangular and hexagonal Cr_7Te_8 morphologies; (e) 2D Cr_7Te_8 thickness distribution.

In XRD analysis, single crystal plane diffraction information is obtained, that is, the (00l) crystal plane diffraction. The peaks of (001), (002), (003), and (004) diffracting planes can be clearly seen from the Fig. 1e, and the corresponding peaks are 14.5°, 29.4°, 44.7°, and 60.9°, respectively, indicating that they are in-plane growth modes. According to the Bragg equation, $2dsin\theta = n\lambda$, the layer spacing of Cr_7Te_8 can be obtained as 6.08A (as shown in Table S1), where λ =1.54056 Å is the X-ray wavelength, d is the spacing of the diffracting planes, and θ is the angle between the incident rays and the diffracting planes.

Table S1. Layer spacing of Cr_7Te_8 calculated via the Bragg equation.

Crystal Plane	θ (°)	d (Å)
001	14.5	6.09
002	29.4	6.07
003	44.7	6.08
004	60.9	6.08
**Average**		**6.08**

From the in-plane and cross-sectional TEM analysis in Fig. 2 and Table S1, the lattice parameters of CVD-grown Cr_7Te_8 can be determined as a = b = 3.90 Å and c = 6.08 Å, which are apparently larger than the value of CVD-grown 1T-$CrTe_2$ [34] and smaller than that of CrTe [35], as shown in the Table S2.

Table S2. Comparison of crystal lattice constants of Cr_7Te_8 nanosheet with 1T-$CrTe_2$ and CrTe.

Materials	Crystal structure	Lattice constant		
		a (Å)	b (Å)	c (Å)
1*T*-$CrTe_2$	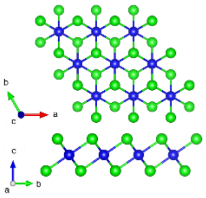	3.77	3.77	6.01
CrTe	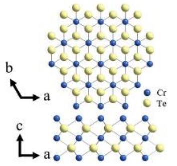	3.98	3.98	6.23
Our Cr_7Te_8	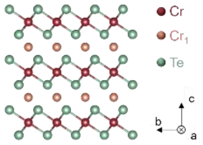	3.90	3.90	6.08

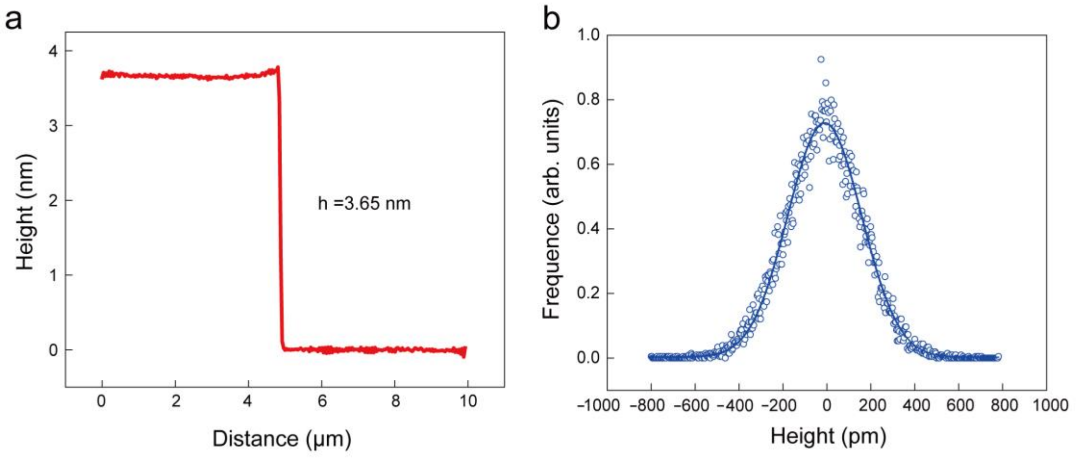

Figure S2. Thickness testing and roughness analysis of Cr_7Te_8 nanosheets: (**a**) The corresponding AFM height profile of the Cr_7Te_8 nanosheet in Fig. 1d, showing a thickness of ~3.65 nm. (**b**) The corresponding surface height analysis of the Cr_7Te_8 nanosheet in Fig. 1d, showing an ultra-small surface roughness of ~0.12 nm.

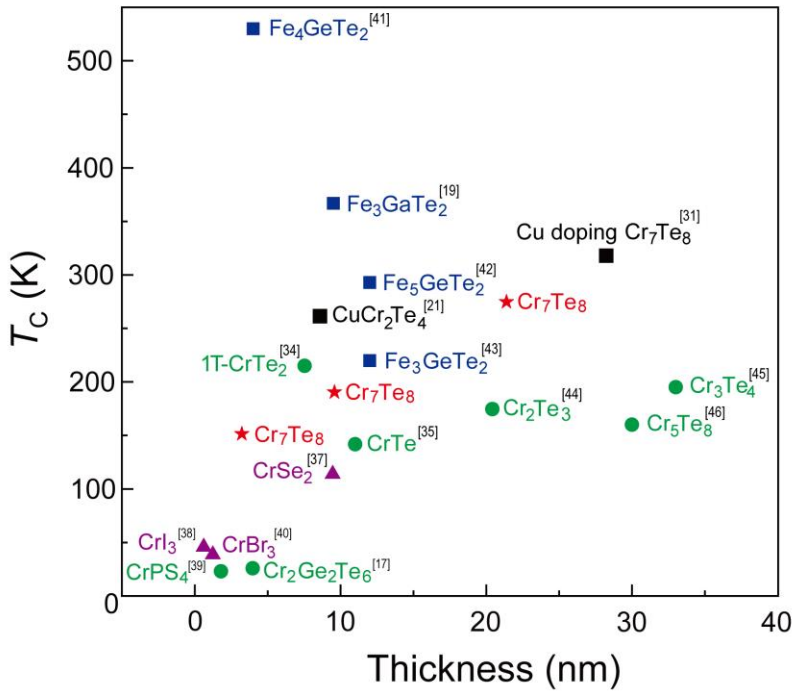

Figure S3. T_C and thickness comparison for various 2D ferromagnet materials.
